# Possible burden of hyperuricaemia on mortality in a community-based population: a large-scale cohort study

**DOI:** 10.1038/s41598-021-88631-8

**Published:** 2021-04-26

**Authors:** Yoichiro Otaki, Tsuneo Konta, Kazunobu Ichikawa, Shouichi Fujimoto, Kunitoshi Iseki, Toshiki Moriyama, Kunihiro Yamagata, Kazuhiko Tsuruya, Ichiei Narita, Masahide Kondo, Yugo Shibagaki, Masato Kasahara, Koichi Asahi, Tsuyoshi Watanabe

**Affiliations:** 1 Department of Cardiology, Pulmonology, and Nephrology, Yamagata University School of Medicine, Yamagata, Japan; 2 Department of Public Health and Hygiene, Yamagata University School of Medicine, 2-2-2, Iida-Nishi, Yamagata, 990-9585 Japan; 3 Steering Committee of Research on “Design of the Comprehensive Health Care System for Chronic Kidney Disease (CKD) Based On the Individual Risk Assessment By Specific Health Checkup”, Fukushima Medical University, Fukushima, Japan

**Keywords:** Medical research, Epidemiology

## Abstract

Hyperuricaemia is a risk for premature death. This study evaluated the burden of hyperuricaemia (serum urate > 7 mg/dL) for all-cause and cardiovascular mortality in 515,979 health checkup participants using an index of population attributable fraction (PAF). Prevalence of hyperuricaemia at baseline was 10.8% in total subjects (21.8% for men and 2.5% for women). During 9-year follow-up, 5952 deaths were noted, including 1164 cardiovascular deaths. In the Cox proportional hazard analysis adjusted for confounding factors, hyperuricaemia was independently associated with all-cause and cardiovascular mortality (adjusted hazard ratios [95% confidence interval]; 1.36 [1.25–1.49] and 1.69 [1.41–2.01], respectively). Adjusted PAFs of hyperuricaemia for all-cause and cardiovascular deaths were 2.9% and 4.4% (approximately 1 in 34 all-cause deaths and 1 in 23 cardiovascular deaths), respectively. In the subgroup analysis, the association between hyperuricaemia and death was stronger in men, smokers, and subjects with renal insufficiency. Adjusted PAFs for all-cause and cardiovascular deaths were 5.3% and 8.1% in men; 5.8% and 7.5% in smokers; and 5.5% and 7.3% in subjects with renal insufficiency. These results disclosed that a substantial number of all-cause and cardiovascular deaths were statistically relevant to hyperuricaemia in the community-based population, especially men, smokers, and subjects with renal insufficiency.

## Introduction

In conjunction with rising physical inactivity and increasing obesity, the prevalence of hyperuricaemia is increasing in the general population^1^. Hyperuricaemia is often accompanied by common disorders such as hypertension, obesity, diabetes, dyslipidaemia, and renal insufficiency, making it difficult to evaluate the significance of hyperuricaemia by itself. Previous epidemiological studies from various countries have shown that hyperuricaemia is independently associated with an increased risk of all-cause and cardiovascular mortality^2–8^, stroke^9^, and coronary heart disease^10^, even after adjusting for the aforementioned risk factors. Similar association was observed in the Japanese general population^11–13^. These observations suggest the significance of hyperuricaemia on life expectancy, independent of other risk factors. However, the burden of hyperuricaemia on life expectancy among the general population with respect to the prevalence of hyperuricaemia is unknown.

In epidemiological studies, to assess the strength of association between exposure to a specific risk factor and the occurrence of a particular event, a measure of relative risk is often used. However, this measure does not consider the prevalence of risk factors; therefore, it is not appropriate to use relative risk to evaluate disease burden. In contrast, the population attributable fraction (PAF) is an integrated measure that includes both the strength of the association of the risk factor with the event and the prevalence of the risk factor among the population. The PAF represents the proportion of a specific outcome in a population, which is relevant to the exposure to the risk factor(s)^14^. In the Japanese population, the PAFs of hypertension^15–20^ and smoking^16,21^ for all-cause and cardiovascular mortality have been reported. However, the possible burden of hyperuricaemia on all-cause and cardiovascular mortality has not been evaluated using the measure of PAF.

To explore this further, we used a national prospective database of 515,979 Japanese annual health check-ups participants from the Japan Specific Health Checkups study (J-SHC study) and assessed the possible burden of hyperuricaemia for all-cause and cardiovascular mortality.

## Results

The subjects’ clinical characteristics at baseline are presented in Table 1. The prevalence of hyperuricaemia was 55,940 (10.8%) (men, 21.8%; women, 2.5%) among a total of 515,979 participants. Hyperuricaemia participants were more likely to be younger, male, smokers, and drinkers and have high blood pressure, diabetes, dyslipidaemia, high BMI, and low estimated glomerular filtration rate (eGFR) compared with those without hyperuricaemia.

**Table 1:** Baseline characteristics of the study population.

	Total participants	Studied subjects	Hyperuricaemia (> 7 mg/dL)	Non-hyperuricaemia (≤ 7 mg/dL)
Number (%)	685,889	515,979	55,940 (10.8)	460,039 (89.2)
Men (%)	42.7	43.0	86.6	13.4
Age (years)	62.3 ± 8.8	62.3 ± 8.9	61.2 ± 9.5	62.4 ± 8.8
Smoking (%)	15.5	15.4	25.6	14.2
Alcohol consumption (%)	46.5	46.5	70.0	43.7
BMI (kg/m^2^)	23.5 ± 3.5	23.5 ± 3.5	25.1 ± 3.5	23.2 ± 3.4
eGFR (mL/min/1.73 m^2^)	76.0 ± 17.3	75.9 ± 17.0	68.4 ± 17.1	76.9 ± 16.7
Hypertension (%)	29.2	29.2	39.1	28.0
Diabetes (%)	13.1	12.2	13.4	12.0
Dyslipidaemia (%)	47.5	47.7	62.5	45.9

Mean ± standard deviation.

*BMI* body mass index; *eGFR* estimated glomerular filtration rate.

During the 9-year follow-up, 5952 (1.2%) deaths were noted (men, 1.8%; women, 0.7%), including 1164 deaths (0.2%) (men, 0.4%; women, 0.1%) owing to cardiovascular causes.

The association between serum urate levels and mortality is shown in Table 2. In the multivariate model adjusted for possible confounders, a significant increase in the hazard ratio (HR) of hyperuricaemia for all-cause and cardiovascular mortality was observed compared with no hyperuricaemia (HR, 1.36 [95% confidence interval {CI} 1.25–1.49] for all-cause mortality and HR, 1.69 [95% CI 1.41–2.01] for cardiovascular mortality). The PAF of hyperuricaemia was 2.9% for all-cause mortality and 4.4% for cardiovascular mortality (approximately 1 in 34 of all-cause deaths, and 1 in 23 of cardiovascular deaths). The PAFs of other common risk factors for all-cause and cardiovascular mortality were 6.7% and 7.6%, respectively, for smoking; 4.9% and 4.7%, respectively, for diabetes; and 3.4% and 11.1%, respectively, for hypertension. In contrast, the HRs for all-cause and cardiovascular mortality owing to dyslipidaemia and alcohol consumption were not increased; therefore, the PAFs of these factors were not calculated.

**Table 2:** Population attributable risk of hyperuricaemia for all-cause and cardiovascular mortality.

	Prevalence (%)	All-cause mortality		Cardiovascular mortality	
		Adjusted HR (95% CI)	PAF (%)	Adjusted HR (95% CI)	PAF (%)
Hyperuricaemia	10.8	1.36 (1.25–1.49)	2.9	1.69 (1.41–2.01)	4.4
Smoking	15.4	1.76 (1.63–1.90)	6.7	1.97 (1.68–2.33)	7.6
Diabetes	12.2	1.68 (1.56–1.81)	4.9	1.63 (1.38–1.92)	4.7
Hypertension	29.2	1.13 (1.07–1.21)	3.4	1.61 (1.40–1.85)	11.1
Dyslipidaemia		0.89 (0.84–0.95)		1.05 (0.92–1.21)	
Alcohol consumption		0.72 (0.68–0.78)		0.69 (0.59–0.80)	
Men (vs. Women)		2.61 (2.42–2.82)		2.55 (2.14–3.03)	
Age (per 1-year increase)		1.06 (1.06–1.07)		1.07 (1.05–1.08)	
BMI (per 1 kg/m^2^ increase)		0.97 (0.96–0.98)		1.00 (0.98–1.03)	
eGFR (per 1 mL/min/1.73m^2^ increase)		1.001 (0.998–1.003)		0.992 (0.987–0.996)	

*HR* hazard ratio, *CI* confidence interval, *PAF* population attributable risk fraction, *BMI* body mass index, *eGFR* estimated glomerular filtration rate.

In the Cox proportional hazard analysis, possible interactions with hyperuricaemia were detected with respect to sex (P < 0.01) and eGFR (P < 0.01) for all-cause mortality and eGFR (P < 0.01) for cardiovascular mortality. Therefore, we performed subgroup analyses of baseline characteristics (Table 3) and found that the PAFs of hyperuricaemia varied depending on the subgroups: as follows 1.3–5.8% for all-cause mortality and 1.6–8.1% for cardiovascular mortality. In particular, the association between hyperuricaemia and death was stronger in men, smokers, and subjects with renal insufficiency (eGFR < 60 mL/min/1.73 m^2^) than in their counterparts. The adjusted PAFs for all-cause and cardiovascular deaths were 5.3% and 8.1% (approximately 1 in 19 all-cause deaths, 1 in 12 cardiovascular deaths), respectively, in men; 5.8% and 7.5% (approximately 1 in 17 all-cause deaths, 1 in 13 cardiovascular deaths), respectively, in smokers; and 5.5% and 7.3% (approximately 1 in 18 all-cause deaths, 1 in 14 cardiovascular deaths), respectively, in subjects with renal insufficiency.

**Table 3:** Subgroup analyses for population attributable fraction of hyperuricaemia.

Subgroups	Prevalence of hyperuricaemia (%)	All-cause mortality		Cardiovascular mortality	
		Adjusted HR* (95% CI)	PAF (%)	Adjusted HR* (95% CI)	PAF (%)
Men	21.8	1.32 (1.20–1.45)	5.3	1.59 (1.32–1.92)	8.1
Women	2.5	2.09 (1.62–2.69)	1.3	2.65 (1.62–4.33)	1.6
< 65 years	11.6	1.34 (1.15–1.55)	2.9	1.71 (1.26–2.31)	4.8
> 65 years	10.1	1.37 (1.22–1.52)	2.7	1.66 (1.34–2.07)	4.0
Hypertension (−)	9.3	1.38 (1.23–1.55)	2.6	2.06 (1.62–2.62)	4.8
Hypertension ( +)	14.5	1.34 (1.16–1.53)	3.7	1.37 (1.06–1.77)	3.9
Diabetes (-)	11.0	1.39 (1.26–1.53)	3.1	1.77 (1.44–2.16)	4.8
Diabetes (+)	12.2	1.25 (1.03–1.51)	2.4	1.44 (0.99–2.09)	3.7
Dyslipidaemia (−)	7.8	1.38 (1.22–1.57)	2.1	1.95 (1.50–2.54)	3.8
Dyslipidaemia (+)	14.2	1.33 (1.18–1.50)	3.5	1.52 (1.20–1.92)	4.9
Smoking (−)	9.6	1.30 (1.17–1.45)	2.2	1.67 (1.35–2.06)	3.9
Smoking (+)	18.0	1.47 (1.26–1.72)	5.8	1.71 (1.25–2.34)	7.5
Alcohol consumption (−)	6.0	1.31 (1.12–1.51)	1.4	1.72 (1.29–2.30)	2.5
Alcohol consumption (+)	16.0	1.39 (1.24–1.55)	4.5	1.65 (1.32–2.07)	6.3
eGFR ≥ 60 mL/min/1.73 m^2^	8.7	1.30 (1.16–1.45)	1.5	1.65 (1.31–2.08)	3.4
eGFR < 60 mL/min/1.73 m^2^	23.9	1.21 (1.04–1.41)	5.5	1.44 (1.09–1.90)	7.3

*eGFR* estimated glomerular filtration rate; *HR* hazard ratio; *CI* confidence interval; *PAF* population attributable fraction; (+), present; (−), absent.

*Adjusted for age, gender, body mass index, smoking, alcohol consumption, eGFR, hypertension, diabetes, and dyslipidaemia.

## Discussion

In this large-scale cohort study, we demonstrated for the first time that a substantial number of all-cause and cardiovascular deaths were statistically relevant to hyperuricaemia, especially in men, smokers, and subjects with renal insufficiency.

In this study, the PAFs for all-cause and cardiovascular mortality among all the subjects were 2.9% and 4.4%, respectively, for hyperuricaemia, and these PAFs were slightly lower than those of smoking, diabetes, and hypertension. To our knowledge, no previous cohort study has reported the PAF of hyperuricaemia for mortality in the general population; therefore, we have no comparison for our results. A previous Japanese study reported that the PAF of hypertension for all-cause mortality was 12% in elderly men and 3% in elderly women, whereas the PAF of hypertension for cardiovascular death was 28% in elderly men and 7% in elderly women^18^. A US report showed that the PAF of diabetes was 3.6% for all-cause mortality and 5.2% for cardiovascular mortality^22^. Because of the differences in age, ethnicity, region of the studied population, and definition of diseases and events, the absolute values of PAF cannot be compared among studies. However, the PAFs of hypertension and diabetes in the current study were similar to those in these previous studies, indicating that our results do not seem to be significantly biased. Considering the current increasing prevalence of hyperuricaemia, the possible burden of hyperuricaemia is also expected to increase.

In the subgroup analyses, hyperuricaemia was independently associated with all-cause and cardiovascular mortality in almost all subgroups even after adjusting for confounding factors. However, the frequency of hyperuricaemia, which differed in each group, greatly affected the PAF. In particular, the PAF in the subgroup of men who had a high frequency of hyperuricaemia was the highest among all subgroups. As the prevalence of hyperuricaemia is increasing in males, the implications of hyperuricaemia is expected to increase in the future. In contrast, the HR of hyperuricaemia for mortality is higher in women than in men, although the prevalence of hyperuricaemia is very low in women. Accordingly, the PAF of hyperuricaemia is low, and its association with mortality in women seems to be limited. Although many studies, including our study, defined hyperuricaemia as serum urate > 7 mg/dL in both men and women, the distribution of serum urate differs between men and women. Therefore, different cut-off values might be more appropriate for both groups.

The PAFs of hyperuricaemia for mortality are also increased in smokers and subjects with renal insufficiency, namely by a factor of 2–3 times higher than that of their counterparts. The precise mechanism is unknown. In addition to high prevalence of hyperuricaemia in these subjects, smoking, renal insufficiency, and hyperuricaemia may have synergistic association with mortality, Based on the current findings, a substantial number of men (21.8%), smokers (18.0%), and subjects with renal insufficiency (23.9%) were at a higher risk for mortality. However, whether all high-risk subjects require urate-lowering medication remains unclear. This should be determined from causality and cost-effectiveness data obtained from intervention trials.

The strength of this study is that we used a large number of samples collected from different regions of Japan, with sufficient events and ability to correct for multiple confounders. Furthermore, various subgroup analyses increased the robustness of the obtained results. However, this study had some limitations. First, serum urate levels were measured only at baseline. Second, although the factors strongly related with serum urate levels were adjusted in this study, the unmeasured confounding factors could exist. For instance, there was no information on medication use for hyperuricaemia and others (e.g., diuretics) in the study subjects. Furthermore, the self-reported life-style factors do not perfectly reflect the actual status. Therefore, the residual confounding factors may be involved in the observed association. Third, the participants were Japanese; therefore, the findings might not be applicable to other populations. Fourth, owing to the observational nature of the study, our findings did not show a direct causal relationship between uric acid levels and mortality. Although this study used the measure of PAF to assess the possible burden of hyperuricaemia, whether hyperuricaemia itself is a direct cause of mortality is undetermined. There is a possibility that the elevation in serum urate is not a cause but a rather reflection of other unmeasured risk factors. Clinical trials utilising interventions to lower urate are necessary to clarify this point.

## Conclusions

This study shows that a substantial number of all-cause and cardiovascular deaths are statistically relevant to hyperuricaemia in this community-based population, especially in men, smokers, and subjects with renal insufficiency. These results indicate that if uric acid levels are causally related to mortality, then lowering uric acid levels may reduce the risk of premature death among a large number of at-risk subjects. However, the causality cannot be determined from this observational study. To verify our findings of the association between urate and mortality, a prospective interventional study using urate-lowering therapies is warranted.

## Methods

### Study population

This study was part of an ongoing “Research on Design of the Comprehensive Health Care System for Chronic Kidney Disease (CKD) based on the Individual Risk Assessment by Specific Health Checkup” study. The study population included participants from the Specific Health Check and Specific Health Guidance, an annual health check-up for all inhabitants of Japan who were aged between 40 and 74 years, covered by the Japanese national health insurance. Between 2008 and 2014, a total of 685,889 subjects, (male, 42.7%; female, 57.3%; age range, 40–74 years) participated in the baseline health check-ups from eight areas: Yamagata, Fukushima, Niigata, Ibaraki, Toyonaka, Fukuoka, Miyazaki, and Okinawa. The details of this study have been described elsewhere^13^. The study was conducted in accordance with the guidelines of the Declaration of Helsinki. The ethics committees of Yamagata University (Approval No. 2008-103) approved this study and waived the need for informed consent from each participant because all data were anonymised before analysis. Among the 685,889 baseline participants, 169,910 (24.8%) subjects (male, 41.7%; female, 58.3%) were excluded from this study because essential data, mainly serum urate levels, were incomplete. Therefore, data of 515,979 subjects (male, 43.0%; female, 57.0%) were included in this analysis. The association between serum urate levels at baseline and all-cause and cardiovascular mortality during the 9-year follow-up (median 3.7 years) was examined, and the PAF was calculated.

### Measurements

The subjects completed a self-reported questionnaire to document their medical history, current medications, current smoking habits (smoker or non-smoker), and current alcohol consumption (drinker or non-drinker). Body weight, height, and systolic and diastolic blood pressure were measured at health check-ups. The presence of diabetes and dyslipidaemia was ascertained by laboratory parameters, including plasma glucose, haemoglobin A1c (HbA1c), triglyceride, low-density lipoprotein cholesterol (LDL-C), high-density lipoprotein cholesterol (HDL-C), and information on medical treatment. Hypertension was defined as systolic blood pressure ≥ 140 mmHg or diastolic blood pressure ≥ 90 mmHg at the health check-up site or the use of antihypertensive medication. Diabetes was defined as a plasma glucose level ≥ 126 mg/dL, HbA1c (NGSP value) ≥ 6.5%, or the use of anti-diabetic medication. Dyslipidaemia was defined as triglyceride levels ≥ 150 mg/dL, HDL-C levels < 40 mg/dL, LDL-C levels ≥ 140 mg/dL, or the use of lipid-lowering medication. Serum urate levels were mainly measured using an enzymatic method. In the Guideline of Japanese Society of Gout and Nucleic Acid Metabolism, hyperuricemia is defined as serum urate levels of more than 7.0 mg/dL^23^. In addition, our previous study using data of the same community-based population, showed that a significant increase in the risk for all-cause and cardiovascular mortality was observed at serum urate levels ≥ 7.0 in men^13^. Therefore, in this analysis hyperuricaemia was defined as serum urate levels > 7 mg/dL. Serum creatinine levels were measured using an enzymatic method, and eGFR was obtained using the Japanese equation^24^. All blood and urine analyses were performed in local laboratories. The methods for these analyses were not calibrated among laboratories; however, the analyses were performed according to the Japan Society of Clinical Chemistry recommended methods for laboratory tests, which have been widely adopted by laboratories across Japan.

### Classification of cause of death

Confirmation of death and classification of the cause of death were performed using the methods previously reported^13^. The incidence of death was monitored annually at the end of 2016, and the causes of death were determined by reviewing death certificates. Death certificates were collected with permission from the Management and Coordination Agency of the Japanese Government. The death code (International Classification of Diseases, 10th Revision) and date of death were reviewed. The cause of death was classified using the International Statistical Classification of Diseases and Related Health Problems 10th Revision (ICD-10) code. Cardiovascular mortality was defined as death due to the circulatory system (ICD-10 codes I00–I99), such as acute myocardial infarction (I21), chronic ischaemic heart disease (I25), cardiomyopathy (I42), heart failure (I50), subarachnoid haemorrhage (I60), intracerebral haemorrhage (I61), and cerebral infarction (I63).

### Statistical analysis

To examine the HRs of hyperuricaemia for mortality, multivariate Cox proportional hazard analyses adjusted for possible confounding factors such as age, sex, smoking habits, alcohol consumption, eGFR, body mass index, hypertension, diabetes, and dyslipidaemia were performed. The assumption of proportional hazards was tested by the Schoenfeld residuals and the graphs of the log–log plot of the relative hazards by time, and it showed no violation of proportionality. PAF was calculated using the formula: PAF = pd × (relative risk − 1)/relative risk, where pd is the proportion of cases exposed to the risk factor^25^. To confirm the association, subgroup analysis was performed on baseline characteristics. The significance of the interaction with confounding comorbidities was tested using multivariate Cox models. Continuous data are expressed as mean ± standard deviation. All statistical analyses were performed using Stata version 14 software (Stata Corp LP, College Station, TX, USA). *P* values < 0.05 were considered statistically significant. In multiple testing in subgroup analysis, *P* value threshold was adjusted using the Bonferroni correction (*P* < 0.05/the number of testing).

## Data Availability

Data cannot be shared publicly due to ethical restrictions on sharing data publicly.
